# Surgery of the Rectum for Practitioners

**Published:** 1913-03

**Authors:** 


**Surgery of the Rectum for Practitioners.** By Sir FREDERICK WALLIS, M.B., B.C., F.R.C.S. Pp. xv, 355. London: Oxford Medical Publications. 1912. 15s. net.

This book is a useful guide to the subject of which it treats. Unfortunately the author did not live to complete the work, though he was correcting the proofs up to within a few days of his death. The book is evidently the work of a surgeon very actively interested in this branch of surgery, and Sir F. Wallis's appointment as surgeon to St. Mark's Hospital gave him the special experience he required in undertaking its production. The book contains many suggestions as to diagnosis and treatment of much practical value, the illustrations are abundant and excellent, and the records of many very interesting cases which were under the writer's own care are to be found in its pages. We must confess that we were rather disappointed in the chapter on the treatment of rectal cancer, but as this comes at the end of the book, probably the author was not at that time able to deal with it in anything like an exhaustive manner.

We presume that the title of the book, which says that it is for practitioners, is intended to indicate that it contains more than a student would be expected to know, and yet that it is not an exhaustive work on the surgery of the rectum. If so, this very well defines the scope of the book, and we feel sure it will be found a very useful help in the diagnosis and treatment of rectal diseases by the general practitioner.

